# Simulation of polymeric mixed ionic and electronic conductors with a combined classical and quantum mechanical model†‡

**DOI:** 10.1039/d2tc05103f

**Published:** 2023-01-31

**Authors:** Alessandro Landi, Maryam Reisjalali, Joshua D. Elliott, Micaela Matta, Paola Carbone, Alessandro Troisi

**Affiliations:** a Department of Chemistry, University of Liverpool Liverpool L69 3BX UK alelandi1@unisa.it A.Troisi@liverpool.ac.uk; b Dipartimento di Chimica e Biologia Adolfo Zambelli, Università di Salerno Via Giovanni Paolo II, I-84084 Fisciano Salerno Italy; c Department of Chemical Engineering, University of Manchester Manchester M13 9PL UK

## Abstract

In organic polymeric materials with mixed ionic and electronic conduction (OMIEC), the excess charge in doped polymers is very mobile and the dynamics of the polymer chain cannot be accurately described with a model including only fixed point charges. Ions and polymer are comparatively slower and a methodology to capture the correlated motions of excess charge and ions is currently unavailable. Considering a prototypical interface encountered in this type of materials, we constructed a scheme based on the combination of MD and QM/MM to evaluate the classical dynamics of polymer, water and ions, while allowing the excess charge of the polymer chains to rearrange following the external electrostatic potential. We find that the location of the excess charge varies substantially between chains. The excess charge changes across multiple timescales as a result of fast structural fluctuations and slow rearrangement of the polymeric chains. Our results indicate that such effects are likely important to describe the phenomenology of OMIEC, but additional features should be added to the model to enable the study of processes such as electrochemical doping.

## Abstract 2

We present a model enabling the simulation of doped polymers in the vicinity of water and ions taking into account large electronic rearrangements coupled with slow dynamics.

## Graphical abstract

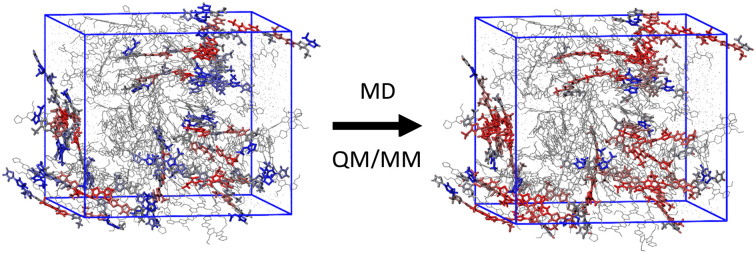

## Introduction

Materials that efficiently couple electronic and ionic charge transport have been recognized as essential in a wide range of technologies:^1–3^ energy storage and generation^4–6^ (batteries and supercapacitors, fuel-cells, water splitting), nanomedicine and healthcare^7–9^ (drug release, pacemakers, cochlear implants, metabolites sensing and control), and other applications^10,11^ (actuators, light-emitting electrochemical cells, ion pumps and neuromorphic computing). Organic materials, which have already been deeply studied for their interesting properties in the field of optoelectronics,^12–16^ are ideal candidates for these applications because of their ease of processing, flexibility, low cost and because their chemical-physical properties can be finely tuned, *e.g.* to ensure perfect integration with cellular tissues for nanomedicine or a light weight for energy storage.^17^

While a growing number of device architectures have been identified in the past years, any organic mixed ionic–electronic conductor (OMIEC) device schematically consists, in essence, of an organic semiconductor (usually a polymer) immersed in an electrolyte solution and connected to one or two electrodes. A change in the voltage experienced by the semiconductor, induced either by electrochemical reactions in the solution, by the detection of an analyte or by an externally applied bias (depending on the specific application) controls the injection of ions into the semiconducting channel, and therefore the doping/dedoping of the material, ultimately inducing a change in the electronic current.

In light of their versatility, several studies focused on this material class, both from an experimental and from a theoretical point of view.^18–21^ Indeed, understanding the impact of several factors (*e.g.* backbone and sidechain design, additives and processing methods) on the polymer morphology and performances would allow to design high-performance OMIEC-based devices.^22^ However, it should be kept in mind that, to achieve this, a static characterization will not suffice, since the OMIEC structure and properties change dynamically in response to external stimuli during the operation of the device.^18,19^

In this respect, molecular dynamics (MD) simulations can provide deep insight on both equilibrium properties (*e.g.* sidechains and backbone conformations, aggregation, *etc.*) and dynamical properties such as electrolyte diffusion, chain motion, and swelling. The structural information obtained from MD makes these simulations an ideal complement to experimental techniques such as X-ray scattering and absorption characterization techniques.^18,23^ Going more into details, several MD studies have been performed focusing in particular on the prototypical blends of poly(ethylene dioxythiophene) (PEDOT), to analyze self-assembly, electronic transport properties,^24–27^ electrolyte-polymer interactions and morphological changes,^28,29^ considering conditions mimicking those of operating devices.^30^ In parallel, another class of OMIECS, *i.e.* conjugated polymers with glycolated side chains, have been recently investigated as an alternative to PEDOT. Various computational studies analyzed the interactions between electrolytes and glycolated (polar) sidechains, disclosing the impact of different anions^31^ and sidechains^32,33^ on ion coordination, chelation, and conductivity.^32,34–36^ Few studies also attempted to reproduce experimental swelling behaviour as a function of the doping level.^37–39^

**Fig. 1:**
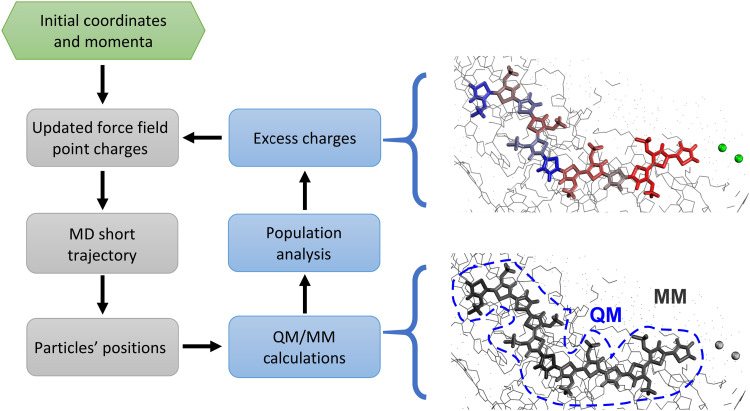
Left panel: QM (blue)/MD (grey) workflow applied in this work. Left panel: Pictorial representation of charge redistributions in an OMIEC. Thick lines represent the QM part, while the spheres represent chlorides. The colour scale indicates the excess charge distribution, ranging from blue (negative charge) to red (positive charge).

Nevertheless, as discussed in ref. 18, classic MD simulations present several issues that currently limit their impact on OMIECs studies. The biggest fundamental obstacle is the overly simplified treatment of electrostatic interaction in classical simulations. Indeed, the semiconducting polymer is immersed in a solvent hosting a significant amount of ionic charges.^40^ Thus, ionic and electronic transport must be assessed together, since they are not independent: ion motion has a significant impact on the charge distribution (Fig. 1, left) and on the electronic states of the chains and *vice versa*. This causes a collapse of the assumptions at the basis of classical atomistic simulations, *i.e.* the Born–Oppenheimer approximation, according to which no change of electronic states occurs during the simulation. In other words, because of the ions’ movement and the highly mobile excess charge on the semiconductor, it is impossible to assign fixed point charges to all atoms; moreover, the charge redistribution during the simulation cannot be described by any polarizable model since the net charge can vary substantially and can be displaced by many Angstroms according to the underlying electronic structure. In other words, using a force field with fixed point charges would result, in practice, in adopting a mean-field approximation, which could potentially lead to errors in the estimation of diffusion properties, ion–ion correlation, and screening effects.^18,41–43^ Therefore, a suitable model should deal with timescales and sizes amenable for classical simulations (*ca.* 10^2^ nm^3^ and 10^2^ ns) while taking into account the electronic structure of the material at the quantum mechanical level of detail, at a reasonable computational cost, with the final goal of using it to determine structure property relationship for a large number of materials, as done in the past for the broader field of organic electronics.^40,44–46^ This could be achieved by creating a workflow where QM/MM calculations of the charges are nested in a loop of MD simulations.^47,48^ Indeed, QM/MM methods have proven to be particularly useful to study electrified interfaces in other contexts, ranging from hydrophobic^49,50^ to graphitic interfaces.^51–53^ In our case, the interface between MD and QM/MM allows the different ionic distribution over time to reflect in an updated classical electrostatic potential experienced by the excess charge over the polymeric chain. Thus, every few MD steps, this effect is taken into account by recomputing the charges at the QM/MM level and then using these updated charges for the next MD run.

**Fig. 2:**
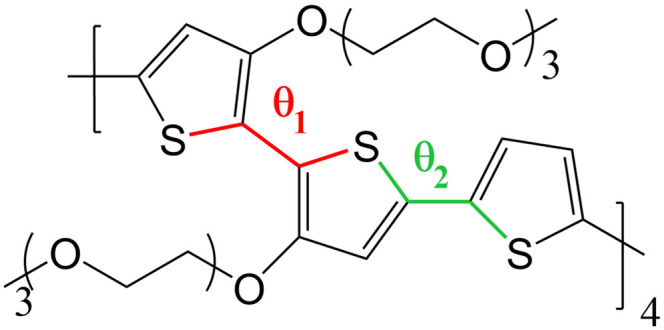
Chemical structure of p(g2T-T) considered in this work, with torsional coordinates *θ*_1_ and *θ*_2_ highlighted.

The objective of this work is to develop such methodology and to illustrate how it can be used to describe a prototypical OMIEC in contact with an electrolyte solution (*i.e.* water and ions). The chosen system is an oligomer model of poly(2,5-bis(3-triethylene-glycoloxythiophen-2-yl)-*co*-thiophene), p(g2T-T), whose structure is reported in Fig. 2. This molecule is representative of the broad class of conjugated polymer backbones with solubilizing ethylene glycol-derived side chains, which has shown to lead to performances similar or higher than the standard PEDOT: PSS^54–56^ with several advantages with respect to the latter system. Indeed, conjugated polymers with ethylene glycol side chains do not have an electrically insulating polyelectrolyte component, thus improving the materials’ capacitance;^54^ moreover, their transport properties are highly tuneable (*e.g.* by using different aromatic cores or side chains) allowing to obtain n-type organic semiconductors^19,57,58^ or electrochemical sensors integrating catalytic enzymes for the detection of biologically relevant metabolites.^59,60^

The methodology is presented in the next section, while the Results section will focus on the characterization of the coupling between electronic and ionic motion, *i.e.* how rapidly and to which extent the excess charge is rearranged within the chain and the spatial correlation between excess positive charge and negative counterions. Most of the conclusion will be drawn from the comparison between simulations where the charge distribution is constant throughout the MD simulation and those where it is updated through QM/MM calculations.

## Methodology

### Classical simulation details

All MD calculations in this paper have been performed using large-scale atomic/molecular massively parallel simulator (LAMMPS) software^61^ in *NPT* ensemble using Nosé–Hoover isothermal/isobaric thermostat and barostat. The force field (FF) used in this study is based on an implementation of All-atom Optimised Potentials for Liquid Simulations (OPLS)^62^ force field. For the neutral chains, equilibrium geometries have been obtained through DFT calculations using the B3LYP functional with the 6-311g** basis set. The atomic point charges for the p(g2T-T), have been obtained fitting the electrostatic potential at points selected according to the CHelpG scheme^63^ as implemented in Gaussian16 software,^64^ while the charges for water, chloride are taken from OPLS.

OPLS torsional potentials have been used throughout, except for the dihedrals between thiophen rings (see Fig. 2) whose torsional potential has been evaluated to reproduce the DFT (B3LYP/6-311g**) torsional potential when all the other non-bonded terms of the interaction (electrostatic and van der Waals) have been included and taking into account the excluded list of the non-bonded interactions in the OPLS force field. The torsional potential is obtainable from the GitHub link reported in the ESI.‡ Water is described by the SPC/E potential, which has been shown to be compatible with OPLS.^65^ Cl^−^ ions, inserted to ensure overall neutrality of the system when charged p(g2T-T) chains are present, are described by OPLS parameters as described in ref. 66. Long-range electrostatics have been taken into account through standard Ewald summation. An integration time step of 2 fs was implemented by imposing an X–H bond constraint on the polymers using the RATTLE algorithm.^67^ Structural analyses on MD trajectories have been performed exploiting the MDAnalysis python library.^68,69^

The initial neutral system, containing 64 tetrameric chains of p(g2T-T) (12 thiophene rings) plus 1624 water molecules, corresponding to 20% weight of the system, has been generated as described in ref. 70. For reproducibility, initial conditions and force field restart files are provided *via* a GitHub repository (see ESI‡).

### Charged polymer system

The study of this class of materials requires a classical description of the excess charge on the polymer which is consistent with the force field of the neutral polymer and that does not require a new definition of the force field as the charge density or polymer chain length are changed. To achieve this goal, we indicate with *q*^FF^_1,*i*_(*t*) and *q*^FF^_0,*i*_ the electrostatic point charges of atom *i* for the charged chain at time *t* and for the neutral chain (time independent), respectively. The electrostatic charges for the charged polymers are computed as a correction to *q*^FF^_0,*i*_:

1 *q*^FF^_1,*i*_(*t*) = *q*^FF^_0,*i*_ + *q*^M^_1,*i*_(*t*) − *q*^M^_0,*i*_

where *q*^M^_1,*i*_(*t*) and *q*^M^_0,*i*_ are the Mulliken charges of the atom *i* for the charged and neutral chain respectively. *q*^M^_1,*i*_(*t*) are computed from a QM/MM scheme (described below) that takes into account the local electrostatic environment of the polymer at a given point of the trajectory, while *q*^M^_0,*i*_ are computed in a reference equilibrium geometry of the isolated polymer chain.

To evaluate the importance of this scheme for distributing the excess charge, we considered an alternative model denoted as FEC (frozen excess charge) where eqn (1) is evaluated at a single geometry (the one optimized in gas), that is used to compute *q*^M^_0,*i*_, and the charge *q*^FF^_1,*i*_ is kept constant throughout the MD trajectory.

### QM/MM calculations

There are clearly many choices to evaluate the Mulliken charges on charged polymers embedded in their local electrostatic environment. The quantum chemical component, to be repeated during the MD simulation and for all the charged chains, can become the rate-determining step and approximate schemes could be desirable. In this work, we opted for calculations of intermediate quality to support future development of more approximated schemes, while avoiding incorrect results due to inaccurate electronic structure calculations. First-principle methods are to be preferred at this stage as semiempirical approaches have not been parametrized for open-shell charged systems. Calculations have been performed at the B3LYP/3-21g* level for each charged polymer chain, including as electrostatic point charges all surrounding atoms.^71^ Since inclusion of periodic boundary condition is not available in our QM/MM code, we resorted to open boundary conditions. To alleviate the error, the simulation box has been translated to have the chain of interest in the centre of the box and all atoms of the simulations are included while preserving the integrity of each molecule to avoid spurious electric fields. Calculations are accelerated more than two-fold by including in the QM part only a truncated –OCH_3_ side chain. We chose B3LYP, the most widely employed functional among the hybrid GGA functionals, since it has been demonstrated to lead to vibronic spectra in good agreement with experimental data also for charged,^72,73^ doped^74^ or radical species,^75^ ensuring that the charge distribution is properly described. Nevertheless, we remark that any further improvement in DFT methods for QM/MM step can be readily included in our workflow.

To improve the efficiency of the algorithm on parallel architectures, the MD steps run on all the allocated CPUs, while several QM/MM calculations of different chains are performed in parallel on different CPUs, *e.g.* 10 QM/MM calculations in parallel with 4 CPUs each, if 40 CPUs are allocated for the run. The process of updating the charges is therefore limited by the slowest electronic structure calculation. The duration of such calculations is more consistent and uniform if the density matrix from the previous calculation is used as a guess and the SCF convergence criterion (RMS of density matrix) is kept to 10^−4^, which is sufficient to achieve a root mean square error on the atomic Mulliken charges of less than 0.015 e with respect to the calculation with tighter convergence.

A critical parameter for this simulation is the time interval between updates of the charges *τ*_CU_. This parameter is clearly system- and temperature-dependent, *e.g.* it should be shorter if ions are more mobile. The timescale for the rearrangement of the excess charge can be validated through the results of the calculation. We have therefore set *τ*_CU_ = 5 ps on the basis of a similar value being used for an electrified interface in ref. 47, and the validity of this choice will be discussed as part of the results. We remark that, in this work, the charges do not move across different chains, but this is not prevented by the methodology itself. In effect, we are capturing the short-time ion-electron dynamics in the interval between chain hopping. The longer the simulation the more important it would be to allow a redistribution of the electronic charge across different chains. This is currently outside the scope of the present work, but it could be implemented introducing a stochastic element where the charge hopping takes place rarely along the MD simulation and changing the potential energy, as done for example in ref. 76.

### Simulation details for the charged system

We have at first generated a system where each oligomer bears a total +1 excess charge. In the initial phase of the system preparation, the excess charge was kept fixed and obtained as described in eqn (1) but using the Mulliken charges of the charged oligomer in vacuum. 64 chloride anions have been inserted randomly to neutralize the excess charge and the system has been minimized (force tolerance = 10^−7^ kcal mol^−1^ Å^−1^) and then equilibrated for 20 ns in *NPT* ensemble, at a constant temperature of 300 K and a pressure of 1.0 atmosphere. Then, the system has been subjected to a simulated annealing by heating from 300 K to 550 K (heating rate = 0.01 K ps^−1^) and this latter temperature has been kept constant for 15 ns to ensure complete randomisation, as the root mean-square displacement of the centre of mass of each chain was computed to be 1 nm in 2 ns.

From this configuration, we have generated two systems where the neutral atomic charges have been restored for some of the oligomers and a corresponding number of chlorides have been removed in order to achieve overall neutrality. We have considered two systems where 16/64 and 48/64 of the chains have total charge +1 (the other chains are neutral) and we will refer to them in the following as 25% charged chains and 75% charged chains, respectively. The systems with the largest amount of charge considered in this work correspond to a doping level close to 30% of the maximum achievable experimentally, based on the value of 0.2 holes per monomer reported in ref. 40 and 77.

These systems have been cooled down to 300 K (cooling rate of 0.02 K ps^−1^) and then subjected to 22 ns of MD simulation with frozen excess charges in *NPT* ensemble at a constant temperature of 300 K and a pressure of 1.0 atmosphere. The first 2 ns have been removed from the analysis to allow equilibration of the trajectory.

Simulations with periodic recomputation of the atomic charge have been performed starting from the last trajectory point computed with frozen excess charge. Some computational considerations are necessary in order to make the whole simulation feasible: with the setup described above, for the system with 75% charged chains, *ca.* 40 steps of MD + QM/MM can be performed per day using a single node with 40 processors (the QM/MM representing roughly the 70% of the total computational time). Using a *τ*_CU_ = 5 ps, this means that roughly 0.2 ns of MD + QM/MM per day can be performed. Such computational consideration limited the length of the trajectories to be performed to 5.1 ns (the first 100 ps of simulation have been removed from the analysis to allow equilibration of the trajectory with variable point charges). This time interval is enough to study the coupling between electronic and ionic motions but currently too slow to study morphological changes. As shown in Fig. S4 in the ESI,‡ using a smaller *τ*_CU_ does not result in significant differences in the charge distribution along the chains, but the computational burden would have prevented to analyze a time window wide enough to gain information about the combined influence of ions and electron movement. In future work, we plan to reduce the computational cost of the QM/MM step and optimize the value of the timestep for charge recomputation. We remark that the system displays rapid (∼10 ps) but very small (∼0.01 e per fragment) charge fluctuations together with a much slower and substantial rearrangement of the charge in the timescale of ion and polymer dynamics. Our model is designed to capture the latter, which is unique of the class of materials.

## Results

### System equilibration with frozen charges

**Fig. 3:**
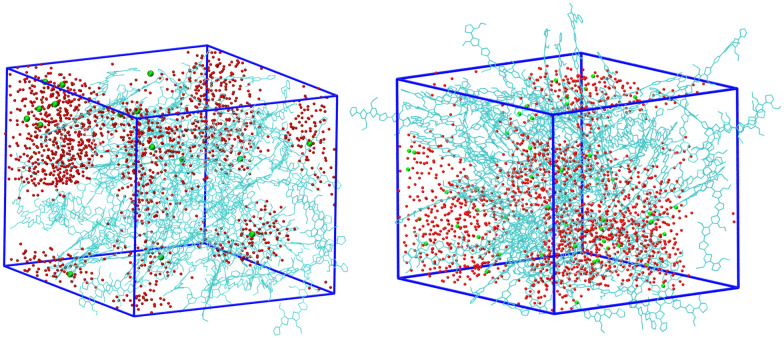
Snapshot of the system with 25% charged chains (left) and 75% charged chains (right). Green points are chloride anions, red points are water molecules (hydrogens and side chains not shown for clarity).

We start the discussion of the results by describing the equilibrated structure obtained for the simulations with frozen charges for the systems with 25% charged chains and 75% charged chains, which constitute our references. Both systems are heterogeneous, and a simple visual inspection of the snapshots (Fig. 3) shows that the majority of water molecules belong to large clusters with several isolated molecules, and the chloride anions are located in the water pockets, as one could have expected. However, we can immediately notice that the water/polymer mixing is better for the system with 75% charged chains. This is expected, since upon increasing the doping level, the Cl^−^ counterions will intercalate in the polymer network to screen the excess positive charges of the p(g2T-T) chains; in parallel, water molecules in the hydration shells of ions will also enter the polymer network.^38,78^

**Fig. 4:**
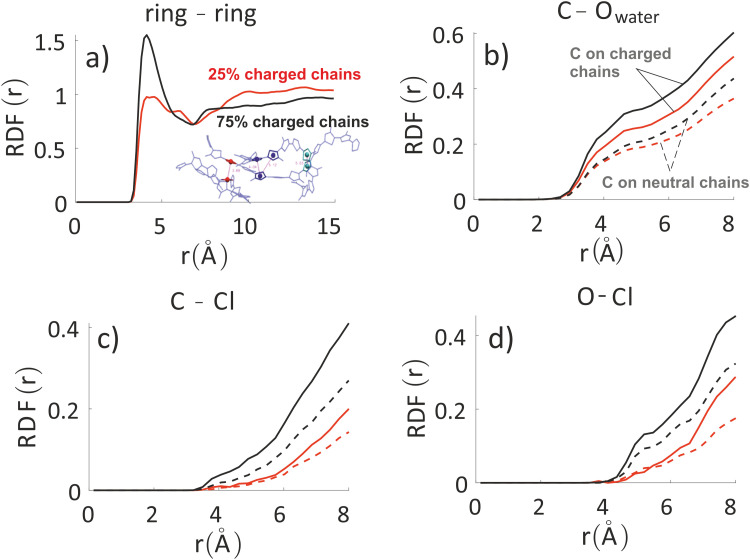
Inter-chain RDF of aromatic rings in the oligomers’ backbones (panel a) for the system with 25% charged chains (red) and the system with 75% charged chains (black). RDF of water oxygens and the C composing the oligomers’ backbones (panel b). RDF of chlorides with C (panel c) and O (panel d) atoms composing the oligomers’ backbones and for the system with 25% charged chains (red) and the system with 75% charged chains (black). Contributions from charged oligomers (solid lines) and from neutral oligomers (dashed lines) have been separated. Note that the last three RDF do not converge to 1 in the range considered, since atom type pairs are prevalently in two separate phases. The full RDF is provided in the ESI.‡

In Fig. 4 we report the radial distribution function (RDF) of the geometric centre of the aromatic rings along the backbone. An inset cartoon is represented in Fig. 4 as an example of rings selection in close interaction. Inspection of the top-left panel in Fig. 4 shows that, maybe surprisingly, there is more inter-chain order for the systems with 75% charged chains despite greater repulsion. This is probably due to the fact that the greater intermixing of the solvent can facilitate ordering, as demonstrated by the fact that the MSD of the rings is almost 1.3 times larger in the latter case over the time interval explored (22 ns). It is worth noticing that the end-to-end distance of the charged chains is 3–4% larger, a small effect in this case that can become important to explain higher order for longer and more highly charged chains in analogy with polyelectrolyte theory.^79^

The other three panels in Fig. 4 (where we have separated contribution from charged oligomers and neutral oligomers) show that, as expected, for both systems the charged chains display stronger interactions with both water and anions, matching the qualitative impression from the snapshots in Fig. 3. More interestingly, the system with 75% charged chains seems to display stronger interactions with both the chloride anions and with the water molecules, when compared with the system with 25% charged chains (remarkably, for both the neutral and charged oligomers). For example, the fraction of water molecules within 7 Å from the aromatic core of the oligomers is 47% and 65% of the total for low and high doping (chain charging) level, respectively. This could be explained considering the higher charge density hosted by the model highly charged system, which leads to stronger repulsions among the (positively charged) polymeric chains, thus creating spaces where water and chlorides can be hosted.

### MD with updated charges

**Fig. 5:**
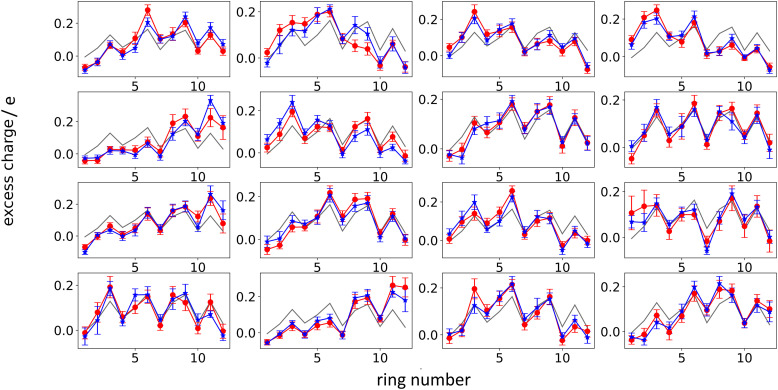
Average excess charge and standard deviation (shown as error bar) for each thiophene ring of the charged oligomers for the 25% charged chains system, in the intervals: 100–300 ps (red); 4.8–5.0 ns (blue). The average excess charge over all the chains has been reported for comparison (grey line). The ring numbering (*x*-axis) follows the structure reported in Fig. 2, going from left to right.

Next, we analyze the results of the model where the excess charge is allowed to redistribute, starting from quantification of such charge redistribution. A first insight on the effect of charge redistribution can be obtained by evaluating the average atomic charge over each thiophenic ring of the charged p(g2T-T) oligomers and the corresponding standard deviation evaluated over 200 ps at different starting times along the trajectory. The results are reported in Fig. 5, where, for comparison purposes, we have also reported the excess charge distribution averaged over all the charged oligomers. Inspection of these figures immediately shows that the charge distribution is quite different from chain to chain; one can compute the standard deviation over the whole time window with respect to the average excess charge over the *i-*th thiophenic ring, *i.e.*, 
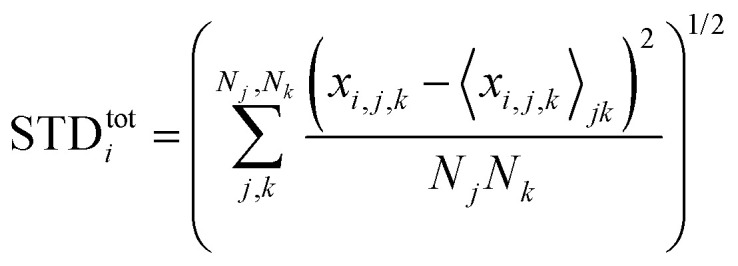
where by *x*_*i,j,k*_ we indicated the excess charge over the *i*-th ring of the *j-*th charged chain at the *k-*th time step, *N*_*j*_ is the total number of the charged chains, *N*_*k*_ the total number of timestep, and 
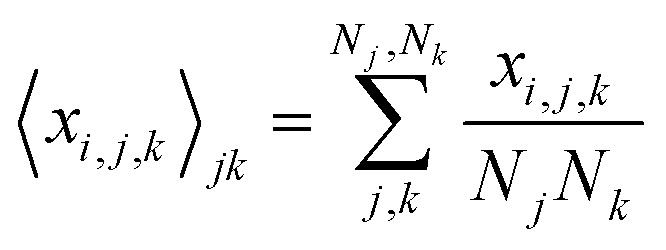
.

We found that, for the 25% charged system, the average STD^tot^ amounts to 0.08 e. This charge displacement is too large to be captured by polarizable centers and the interaction between chains cannot be described accurately by mean-field studies assuming a constant charge distribution as the error in the electrostatic interaction energy exceeds the thermal energy.

Another interesting aspect is the standard deviation of the charge over each thiophenic ring 
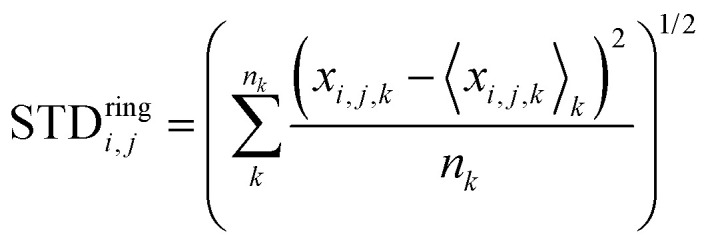
evaluated over a smaller number of timesteps *n*_*k*_ (shorter time window). For example, over 200 ps STD^ring^ is on average 0.03 e, likely due to the fluctuation of the local electrostatic potential, but not too important for the dynamics. The changes over 5 ns are also relatively small and only marginally larger (0.04 e). Occasionally, however, there are more substantial rearrangements of charge (see Table 1), thus suggesting that the charge update cannot be neglected, but maybe can be performed less frequently.

**Table 1:** Average and maximum standard deviation (STD^ring^) of the total charge on each thiophenic ring belonging to a charged chain for the two systems under study evaluated over the interval 100–300 ps or over the whole time spanned by the MD + QM/MM simulation

	25% charged chains	75% charged chains
100–300 ps		
Maximum STD^ring^	0.074	0.077
Average STD^ring^	0.029	0.029

100–5000 ps		
Maximum STD^ring^	0.082	0.193
Average STD^ring^	0.036	0.046

Given the strong difference between the charge distributions of the oligomer in the reference geometry and the various oligomers sampled during the MD, we have performed an analysis (see ESI‡) to assess whether this difference is due to the different conformation assumed by the oligomers during the MD run or rather due to the different electrostatic environment experienced. The results, reported in Fig. S3 (ESI‡), show that probably both factors have some influence, since different chains have different charge distributions even when the environment is neglected. Nevertheless, the main factor influencing the charge distribution is clearly the different environment. In particular, the variance of charge due to conformational changes only is about ten times lower than the variance of charge found during the simulations where both the different environment and the different conformation are taken into account.

We have performed the same analysis also for the system with 75% charged chains, obtaining an average STD^tot^ of 0.11 e. Moreover, we have reported in Table 1 the maximum and the average STD^ring^ for the charge fluctuation evaluated over each thiophenic ring of all the charged chains, over two time intervals, *i.e.* 100–300 ps and 100 ps–5 ns, for both the 25% charged chains and the 75% charged chains system. It is easy to see that the system with 75% charged chains has quite higher maximum standard deviation, *i.e.* there is a somewhat larger fluctuation of the charges during the simulation. The implications of these results are two-fold. First of all, from a methodological point of view, this suggests that a mean-field approximation becomes less adapt for the description of these systems when the doping level increases. Secondly, this analysis suggests that hole–hole interactions are more important in determining the localization of the excess charge than hole–anion (chlorides) interaction.

**Fig. 6:**
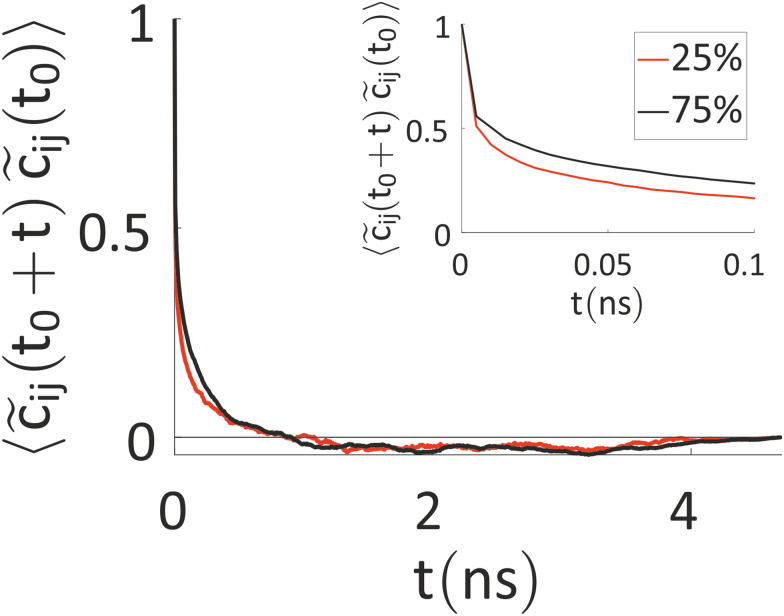
Autocorrelation function of *c̃*_*ij*_(*t*), averaged over the rings of all the charged chains for the system with 25% (red) and 75% (black) charged chains. In the inset, a zoom with the first 100 ps is reported.

Our results indicate that the charges show a fluctuation over time, thus ruling out a mean-field approach, but this variation is somewhat slow. Thus, we have tried to better characterize the timescale of charge reorganization (*e.g.* determining how frequently the charge needs to be updated) a feature that is critical for the development of more efficient methods and to understand the degree of coupling of nuclear motions and excess charge. To this end, we have evaluated the autocorrelation function (ACF) of *c̃*_*ij*_(*t*) = *c*_*ij*_(*t*) − 〈*c*_ij_〉_*t*_, where *c*_*ij*_(*t*) is the charge on ring *i* of chain *j* at the time *t*, while 〈*c*_*ij*_〉_*t*_ is the time average of this quantity over the simulation. The result, averaged over the rings of all the charged chains, is reported in Fig. 6.

The timescale over which the ACF decays is of the order of nanoseconds, which is similar to the timescale for the ions’ movement^80^ (the latter is set by the diffusion coefficients of the ions, discussed below). Interestingly, this timescale rules out the feasibility of first-principles-based molecular dynamics (FPBMD). Indeed, FPBMD is in principle able to capture the fluctuation of point charges which is brought about by the presence of the electrolyte solutions; however, because of its high computational cost, it is typically restricted to short dynamical trajectories, on the order of hundreds of picoseconds, where only several hundred water molecules and unrealistic low ionic concentrations are considered.^81,82^ The slow evolution of these heterogeneous systems, seen for example by changes in monomer charge of tens of electronic charge in 5 ns, indicates on the contrary that further methodological advances are necessary to extend the computational observation window to longer timescales.

**Table 2:** Evaluated diffusion coefficients for chlorides and water in the systems under investigation. We have categorized water molecules in two categories as explained in the text. In parentheses, the number of water molecules in the two classes is reported. The error of the mean diffusivity was estimated from the standard deviation of diffusivity across molecule

	25% charged chains	75% charged chains
Cl^−^ (diffusion coefficient × 10^9^ m^−2^ s^−1^)		
FEC	0.61 ± 0.06	0.37 ± 0.02
MD + QM/MM	0.54 ± 0.06	0.34 ± 0.02

H_2_O (diffusion coefficient × 10^9^ m^−2^ s^−1^)		
FEC-interface	1.03 ± 0.02 (768)	0.684 ± 0.006 (1053)
FEC-bulk	1.21 ± 0.01 (856)	0.738 ± 0.006 (571)
MD + QM/MM-interface	0.93 ± 0.02 (791)	0.584 ± 0.008 (1065)
MD + QM/MM-bulk	1.17 ± 0.01 (833)	0.699 ± 0.008 (559)

### Comparison of FEC and MD + QM/MM model

**Fig. 7:**
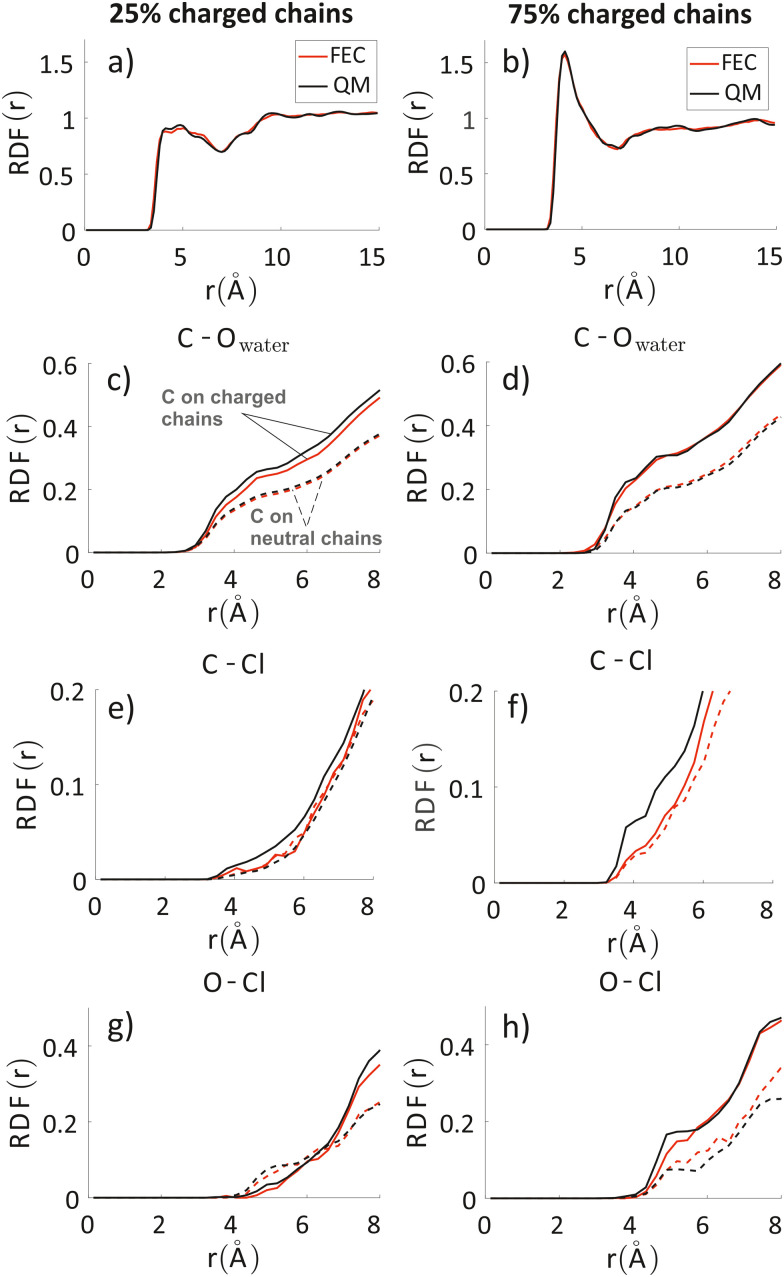
Inter-chain RDF of aromatic rings in the oligomers’ backbones (panels a and b); RDF of water oxygens and the C composing the oligomers’ backbones (panels c and d). RDF of chlorides with C (panels e and f) and O (panels g and h) atoms composing the oligomers’ backbones. Left column: system with 25% charged chains; Right column: system with 75% charged chains. Red lines refer to FEC approach, black lines to MD + QM/MM (in the legend reported as “QM” for simplicity) approach. Contributions from charged oligomers (solid lines) and from neutral oligomers (dashed lines) have been separated. The RDFs with an expanded range are provided in the ESI.‡

To analyze any difference between the structures obtained through FEC approach and MD + QM/MM approach, we have compared the RDF of the aromatic rings along the backbone and the RDF of different atoms composing the oligomers’ backbones with chlorides and water molecules. The RDF of the aromatic rings (top panels in Fig. 7) shows that the polymer–polymer interactions are virtually unchanged between the two approaches, probably also because the length of the updated-charge simulation is too short to see appreciable evolution of this aspect. This is also reflected by the similarity of the RDFs of the oligomers’ backbones with chlorides and water molecules for high distances (*r* > 10 Å, see Fig. S2 in the ESI‡). For that reason, in Fig. 7, we focus on the short-range interactions (*r* < 8 Å) between ions and oligomers.

Inspection of the RDF of C–O(water) shows little or no changes between FEC and MD + QM/MM approaches, indicating that the water dipoles are not enough to induce charge reorganization; this is also related to the virtually unchangeable morphology in the limited observation window, as already discussed. On the contrary, the short-range interactions between Cl^−^ and C are substantially changed in the MD + QM/MM approach, in particular for the 75% charged chains system. In particular, we can notice that the difference between charged and neutral chains is enhanced in the MD + QM/MM approach, resulting in a much larger average concentration of the anion near the charged chains at van der Waals distance: within 5 Å from any C atom in a charged chain the concentration of chloride is 2.2 and 2.6 times higher for the system with 25% and 75% charged chains, respectively.

### Water and chloride diffusivity

We have evaluated diffusivity from the mean square displacement (MSD) from a starting time *t*_*k*_ for a time interval *t* and averaging over *N*_T_ starting times and *N* particles *i*:2
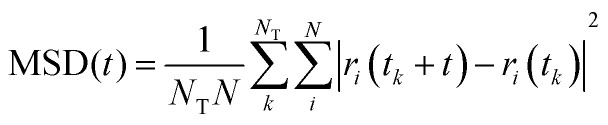


In homogeneous systems, the diffusivity can be computed^83^ as 
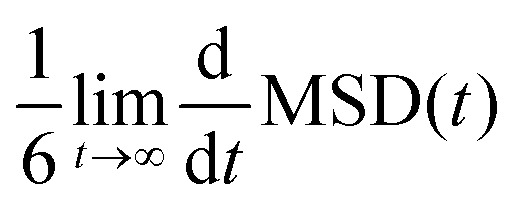
, but, in this heterogeneous system, the diffusivity does not converge to a constant value by increasing the time interval, because water and chlorides occupy a limited region of space and their diffusivity across the polymer phase is much slower. We, therefore, report a short-time diffusivity at 100 ps computed as3
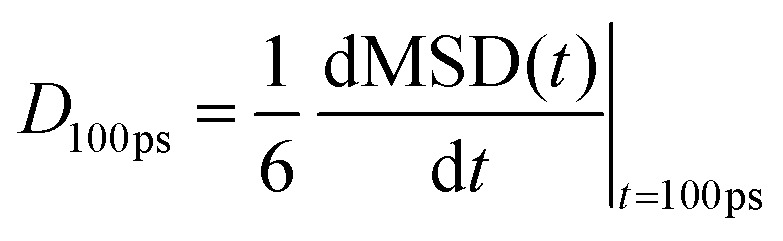


In particular, we have evaluated separately the water diffusivity for water molecules near the polymer (*i.e.* closer than 7 Å from the backbone of pg2T-T at time *t*_*k*_) or far from the polymer (*i.e.* distances higher than 7 Å). We have chosen to study separately the behaviours of these two classes since water molecules closer to the polymers are characteristic of the region involved in the hydration process, while “far” water is expected to be less influenced by the presence of the polymer. Indeed, see Table 2, this latter class shows a higher diffusion coefficient, more resemblant of the behaviour of isolated water (SPC/E diffusion coefficient for bulk water is 2.75 × 10^−9^ m^2^ s^−1^).^84^ When comparing the diffusion coefficients for the systems with 25% or 75% charged chains, we notice that the latter has lower diffusion coefficients for both water and chlorides. This is probably due to the better solvent dispersion in the polymeric phase at increasing doping level (see also Fig. 3): diffusion is reduced near the interface, as the accessible volume for each molecule is reduced, in agreement with what was found with other electrified interfaces.^47^ Stronger fluctuation of the electrostatic potential in more highly charged system can also contribute to a reduced diffusivity of charged or polar species.

Table 2 also shows that the diffusion of bulk water displays, expectedly, only little variations passing from FEC to MD + QM/MM models. Conversely, water molecules closer to the polymer have indeed different diffusion coefficients using FEC or MD + QM/MM approach, because they are more affected by the atomic charges (frozen rather than updated during the MD) of the chains. The water diffusivity is reduced by the effect of updating the excess charges likely because of stronger interactions with the mobile charge on the polymer. This suggests that updating atomic charges may be important when studying the hydration process of OMIECS materials. The results for the anions seem to follow a similar trend but the degree of confidence for such conclusion is lower because of the limited statistics (*i.e.* there are much fewer anions than water).

### Particle-hole RDF

As a final analysis, we have introduced a particle-hole (excess positive charge on polymer) RDF as a generalization of the conventional RDF:4
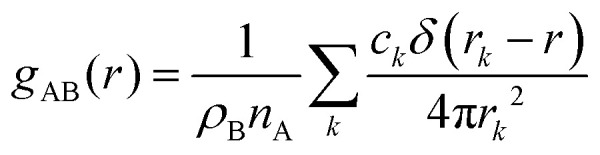
Where *ρ*_B_ is the number density of particle B, *c*_*k*_ is fractional charges on site *k* and 
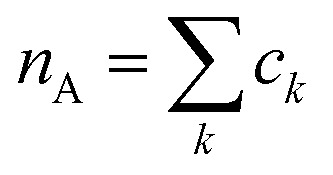
 the total charge. *r*_*k*_ is the distance between site *k* and any particle B. The fractional charge is identified with the excess charge on the charged oligomers, while particle B in this case is the anion. The function expresses the probability of a certain amount of excess charge to be at a certain distance from the anion and it is useful to describe the charge delocalization in a “semiclassical” simulation.

**Fig. 8:**
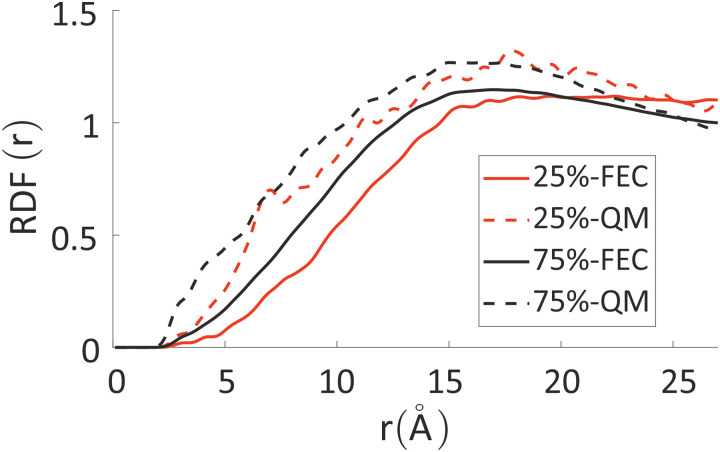
RDF between chlorides and all the other atoms in the system, weighted for their fractional excess charge, see eqn (4). Solid lines refer to FEC approach, while dashed lines to MD + QM/MM approach.

The differences between the simulations with FEC approach or MD + QM/MM approach are remarkable at short distances (<10 Å) and, as expected, they become irrelevant at greater distances. The anions draw excess charge closer to them and such charge can be computed from the integrated excess charge within a distance *L* as 
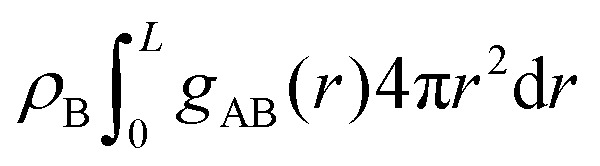
. Considering the case with 25% charged chains and within a distance *L* = 6 Å, the excess charge near the anion is 0.13 e for the case with updated charges and just 0.04 e for the case with frozen charges. The difference is only slightly reduced for the case with 75% charged chains, where we expect some greater electrostatic screening. One of the challenges in the modelling of electrified interfaces^85^ with soft materials is to describe the initial steps of charging, which increases the interaction between the charged polymer and the counterions and facilitates the formation of more mixed organic/water phase. While the interaction described by Fig. 8 offers insight on the initial steps of polymer charging, the time window achievable through these simulations does not allow to study the much slower process of water–polymer mixing.

## Conclusions

This paper presented a study of the coupling between ionic and electronic motion in polymeric semiconductor in close contact with a solution of electrolytes. The focus of this study has been the characterization of the timescales for the dynamic rearrangement of excess charge on polymers as a result of the motion of the charged polymer chain and the surrounding polymer molecules, water and ions. Considering a prototypical interface encountered in these types of materials, we constructed a scheme to evaluate the classical dynamics of nuclei while allowing the excess charge of the polymer chains to rearrange following the external electrostatic potential. This has been achieved with an ad-hoc coupling of QM/MM calculations and classical MD simulations.

One of the main findings is that the rearrangement of charge is substantial (tenth of electronic charge over the length of many Angstroms) and cannot be captured by a mean-field description of the excess charge or polarizable dipole moments. This is due in large part to the interaction with other charged chains in the organic phase but also to the interaction with ions/water and the conformation of the polymeric chain.

The timescale for the dynamical rearrangement of the excess charge on the polymer is shown to be extremely challenging for computation. The charges on each atom fluctuate rapidly, with a characteristic timescale of picoseconds, but the overall time evolution of the charge distribution on a chain is relatively slow (the standard deviation of the charge on each ring is around 0.03 e over 200 ps for both systems studied). However, if the system is observed for longer, large charge rearrangements are seen on individual chains in timescales of several nanoseconds, a time window at the limit of the current methodology. The first direction of future works is the extension of the window spanned by computational study to reach the hundreds of ns range, possibly ignoring the short timescale fluctuation of atomic charge, lowering the accuracy (increasing the speed) of the QM component used in this work, or incorporating our model in a coarse graining approach, such the ones discussed in ref. 86–88. Indeed, we have seen evidence of different and specific interactions between charged chains and anions that may be critical to understand the process of electrochemical charging of such polymers as well as significant differences in the behaviour of water at the interface with the polymer in the presence of mobile charges.

It should also be remarked that the phenomenology of OMIECs is quintessentially multiscale, not only because the motion of charge carriers within a few picoseconds is coupled with the motions of nuclei in the nanosecond range. Indeed, if the system is observed for longer, one should include the effect of charge transfer across polymer chains which has been ignored here.

## Conflicts of interest

There are no conflicts to declare.

## Supplementary Material

TC-011-D2TC05103F-s001
