# Supplementary material for: Simulation of polymeric mixed ionic and electronic conductors with a combined classical and quantum mechanical model
Source: J Mater Chem C Mater. 2023 Jan 31;11(24):8062–73. doi: 10.1039/d2tc05103f (PMC10286221; doi:10.1039/d2tc05103f)
Supplement: TC-011-D2TC05103F-s001 [file TC-011-D2TC05103F-s001.pdf]

Electronic Supplementary Material (ESI) for Journal of Materials Chemistry C.
This journal is © The Royal Society of Chemistry 2023

Supporting Information for:

Simulation of polymeric mixed ionic and electronic conductors with a combined classical and quantum mechanical model

Alessandro Landi*,[a,b] Maryam Reisjalali,[a] Joshua D. Elliott,[c] Micaela Matta,[a] Paola Carbone[c] and Alessandro Troisi*,[a]

[a] *Department of Chemistry, University of Liverpool, Liverpool L69 3BX, United Kingdom*

[b] *Dipartimento di Chimica e Biologia Adolfo Zambelli, Università di Salerno, Via Giovanni Paolo II, I-84084 Fisciano (SA), Italy*

[c] *Department of Chemical Engineering, University of Manchester, Manchester M13 9PL, United Kingdom*

* alelandi1@unisa.it , A.Troisi@liverpool.ac.uk

GitHub Repository

At the link https://github.com/XelaleX1/MD_QM_paper, we provide LAMMPS input, torsional potential for the dihedrals highlighted in Fig. 2 of the main text and starting topologies for the systems under study.

Full RDF for the systems under study

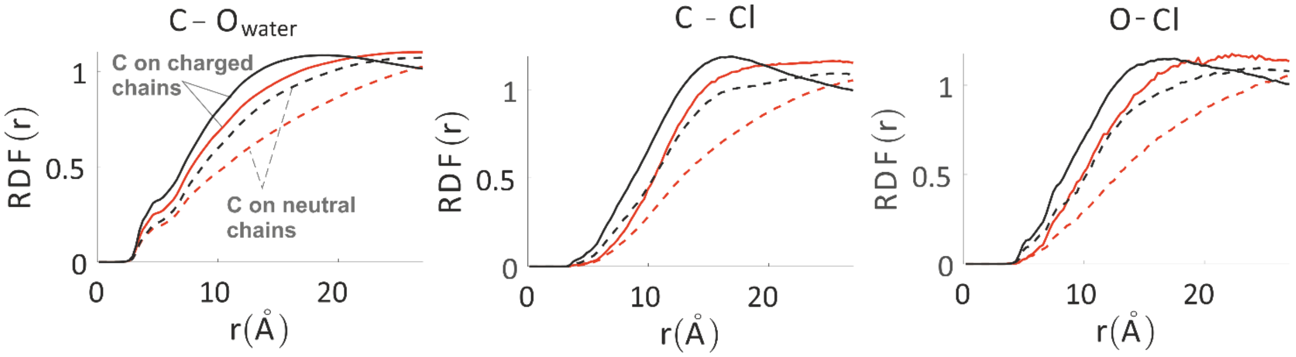

Figure S1: RDF of water oxygens and the C composing the oligomers' backbones (left). RDF of chlorides with C (middle) and O (right) atoms composing the oligomers' backbones and for the system with 25% charged chains (red) and the system with 75% charged chains (black). Contributions from charged oligomers (solid lines) and from neutral oligomers (dashed lines) have been separated.

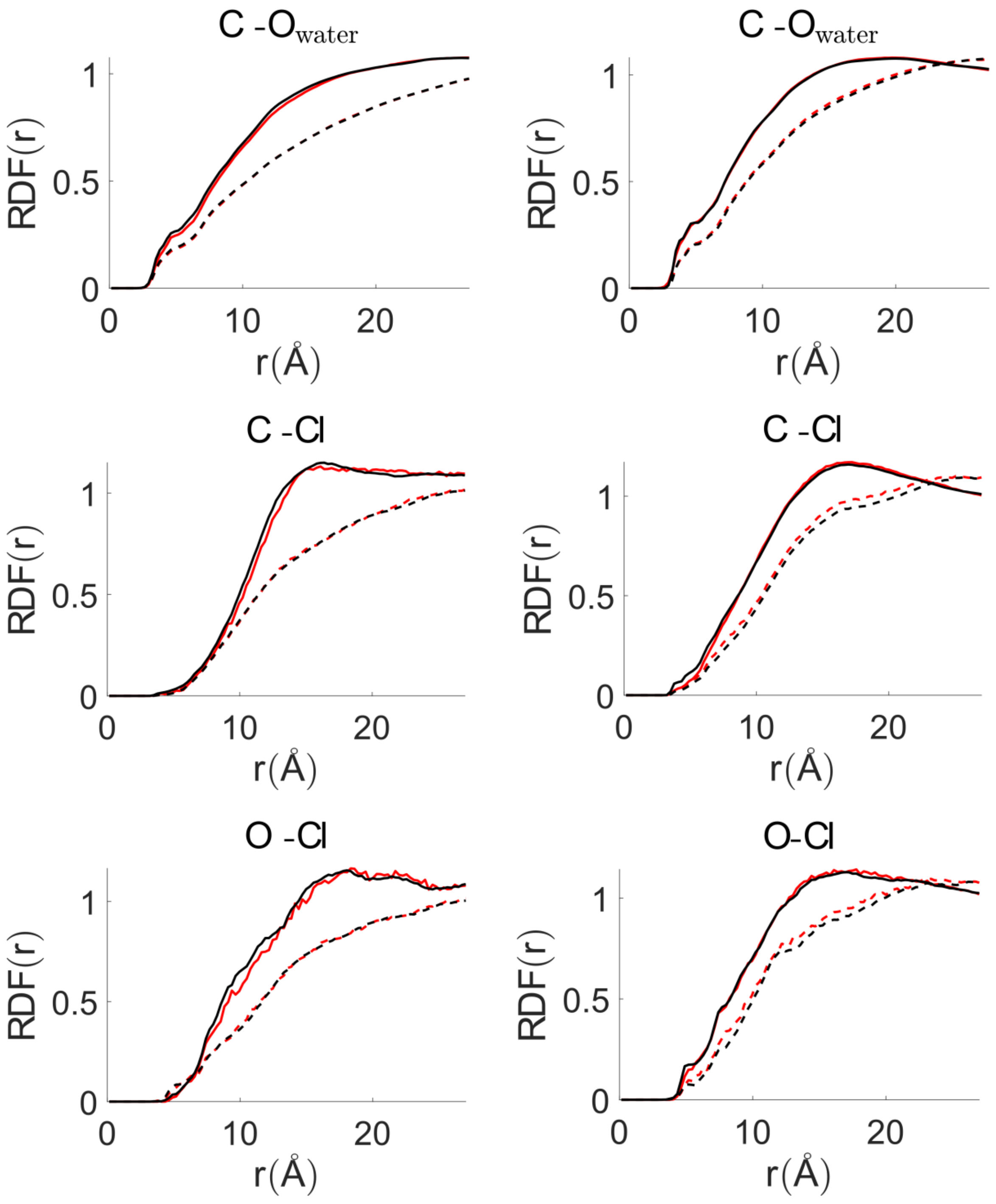

Figure S2: RDF of water oxygens and the C composing the oligomers' backbones (Top) RDF of chlorides with C (Middle) and O (Bottom) atoms composing the oligomers' backbones. Left column: system with 25% charged chains; Right column: system with 75% charged. Red lines refer to FEC approach, black lines to MD+QM/MM (in the legend reported as "QM" for simplicity) approach. Contributions from charged oligomers (solid lines) and from neutral oligomers (dashed lines) have been separated.

Influence of conformation and environment on charge distribution

To check if the different charges distribution is due to different chain conformation assumed by the chains during the MD run or rather to the different environment experienced, we have evaluated the Mulliken charges distribution along all the charged chains for a sample snapshot (after 2 ns) taking into account the environment as point charges (see Methodology section in the main text) or conversely neglecting the environment, i.e. considering the isolated chains. The results, reported in Fig. S3, show that probably both factors have some influence, since different chains have different Mulliken charges distribution even when the environment is neglected (blue lines in the various panels). Nevertheless, the main factor influencing the charge distribution is clearly the different environment, as demonstrated by the significant difference between the red and blue lines.

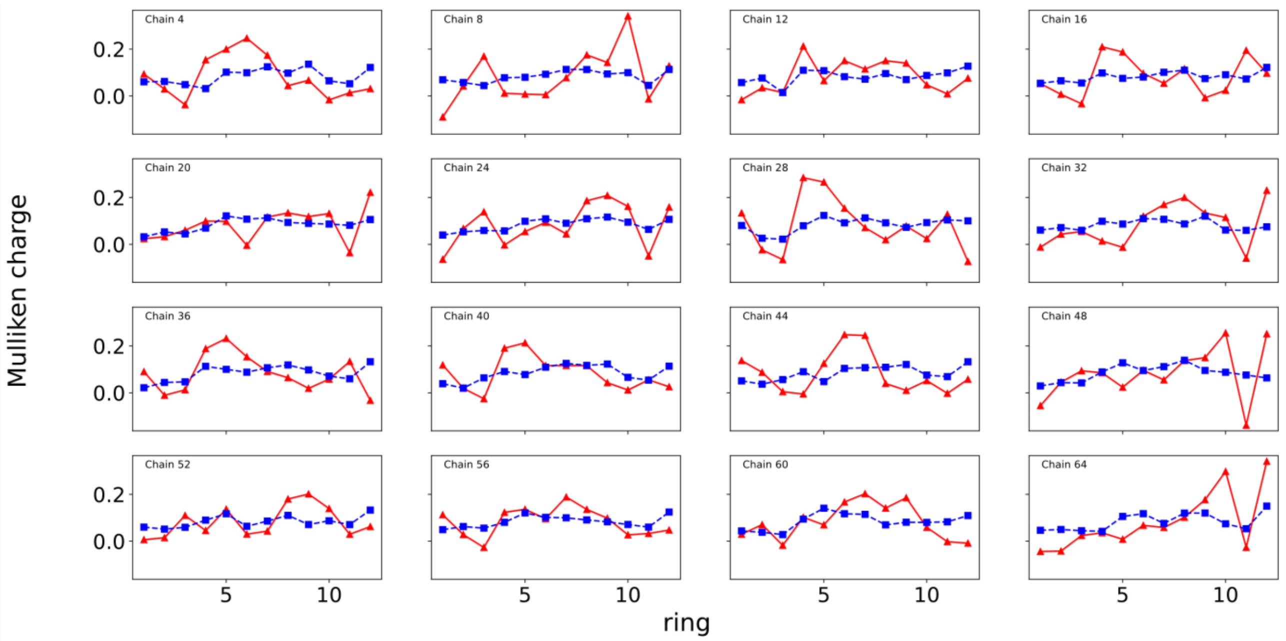

Figure S3 Mulliken charge for each ring of the system with 25% charged chains after 2 ns for the chain with surrounding point charges (red) or neglecting the environment (blue).

Influence of τ_{CU}

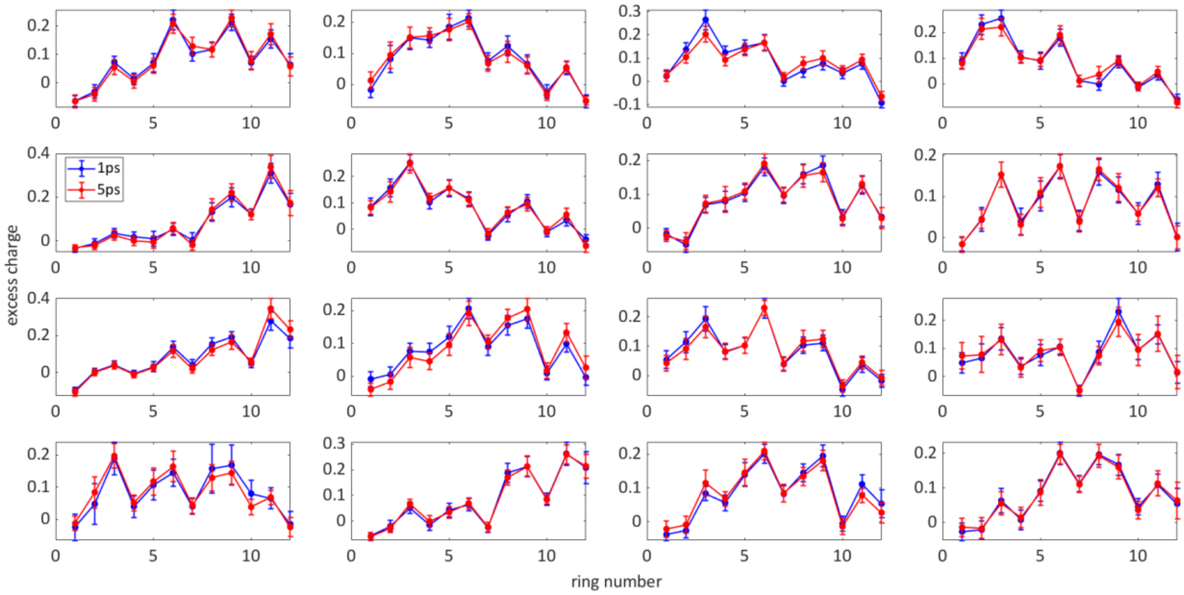

Figure S4 Average excess charge and standard deviation (shown as error bar) for each thiophene ring of the charged oligomers for the 25% charged chains system, in the interval 5-5.2 ns, using a τ_{CU} = 1 ps (blue) or τ_{CU} = 5 ps (red).
